# A rapid assessment of Uganda police force and Uganda prisons service health facilities capacity to roll out the virtual elimination of mother to child HIV transmission (eMTCT) strategy

**DOI:** 10.1186/1471-2334-14-S2-P28

**Published:** 2014-05-23

**Authors:** Lillian Ayebale, Benjamin Lutimba, Erasmus Tanga

**Affiliations:** 1 Research Triangle Institute International, SPEAR Project, Kampala, Uganda; 2 World Vision Uganda, SPEAR Project, Kampala, Uganda

## Background

Uganda has embraced the strategy of virtual elimination of mother to child HIV transmission (eMTCT) by 2015 which has seen the roll out of Option B + country wide. According to the Modes of Transmission report 2009, HIV prevalence among uniformed personnel was 18.4percent, a rate that is almost three times the national average. However little has been done to reach Uganda police Force (UPF) and Uganda Prisons Service (UPS) communities, most affected by HIV and AIDS. In October 2012, The Supporting Public Sector workplaces Expand Action and Responses to HIV (SPEAR) Project funded by USAID, planned to implement eMTCT interventions in the health facilities (HF) of UPF and UPS.

## Objective

This was an assessment to determine the capacity of UPFs and UPS HF to roll out the eMTCT strategy.

## Methods

Thirty four Prisons and 23 police HF were listed for assessment to offer comprehensive Artiretroviral Therapy and roll out the eMTCT strategy. A standard Ministry of Health (MOH) ART accreditation assessment tool, which looked at human resource, equipment and supplies, physical space, and additional HF inputs was used. The assessment team, comprised of MOH Officials, UPF and UPS representatives, and SPEAR project staff, administered the questionnaire to HF staff, inventoried equipment, supplies, and registers for each HF.

## Results

Nineteen HFs (10 UPS, 9 UPF) met the inclusion criteria for the ART accreditation assessment; 17 HCIII, 1HCIV and 1Hospital. Only 1/10 of Prison and 1/9 of Police facilities had at least one medical doctor and had been accredited as ART centers but did not meet the other minimum requirements as per the national guidelines for eMTCT roll out. 9/10 prisons and 3/9 of police had at least one clinical officer. 60% of all the facilities assessed had a midwife. 7/10 prisons facilities had limited space for Antenatal Care Clinic and 8/9 of police facilities had no basic equipments and supplies, lacked physical space and functional laboratories.

## Conclusions and recommendations

HFs faced common challenges impeding the rollout of eMTCT including; inadequate skilled staff, lack of usable space for a fully functional clinic, limited laboratory and medical equipment, and inadequate financial commitment for providing necessary upgrades. The assessment reflected a great need for health systems strengthening not only to roll out eMTCT but for general healthcare.

